# Eating and weight related cognitions in people with Schizophrenia : A case control study

**DOI:** 10.1186/1745-0179-2-29

**Published:** 2006-10-31

**Authors:** Yasser Khazaal, Emmanuelle Frésard, Grégoire Zimmermann, Nathalie Morinière Trombert, Valentino Pomini, François Grasset, François Borgeat, Daniele Zullino

**Affiliations:** 1 Department of Psychiatry – CHUV, University of Lausanne, Site de Cery, 1008 Prilly, Switzerland; 2 Institut Universitaire de Psychothérapie, Département de Psychiatrie-CHUV, Université de Lausanne, Site de Cery, 1008 Prilly, Switzerland; 3 Chaire de psychologie clinique, Département de Psychologie, Université de Fribourg, Fribourg, Switzerland; 4 Fondation Edith Seltzer, Médical Center Chantoiseau, Briançon, France; 5 University Hospitals of Geneva, Division of Substance Abuse, Rue Verte 2, 1205 Geneva, Switzerland

## Abstract

**Background:**

Patients with antipsychotic-induced weight gain (WG) regularly report on unsuccessful dietary trials, which suggests strong biological weight gain drive that is extremely hard to overcome with thoughts, such that behaviour doesn't change despite some intent to change. The purpose of the present study was to assess cognitions specifically related to restrained eating in severely overweight patients with schizophrenia treated with antipsychotic drugs.

**Methods:**

Forty outpatients with schizophrenia and 40 controls without psychiatric disability were included. Both groups were composed of one subgroup severely overweight (defined as a BMI > 28), and a comparison sample (BMI<28). The revised version of the Mizes Anorectic cognitive questionnaire (MAC-R) was used in this cross-sectional case-control study.

**Results:**

Gender was significantly related to eating disorders cognition, women scoring higher than men. Patients with schizophrenia in general scored higher on the MAC-R total scale and on the MAC-R subscale 2, the latter score representing rigid weight regulation and fear of weight gain. When comparing the two groups of subjects with BMI < 28, it appeared that patients with schizophrenia also scored higher on MAC-R total scale, the subscales 2 and 3, the latter subscale 3, indicating altered self control and self-esteem.

**Conclusion:**

As is the case in weight gain of subjects without schizophrenia, the present results suggest that the cognitive distortions, as assessed by the MAC-R, may play an important role in weight gain also in patients with schizophrenia, and in weight gain associated with antipsychotic pharmacotherapy. Particular attention to these processes may help to improve the management of antipsychotic drugs induced weight gain.

## Background

Antipsychotic drugs (AP) induced weight gain (WG) under chronic administration occurs in up to 50 % of patients [1] and can lead to central or abdominal obesity [2-5].

Antipsychotics vary in their propensity to cause weight change with long-term treatment, and some of the atypical antipsychotics are among the drugs most frequently associated with this side effect [1,6]. Besides the aesthetic inconvenience, being overweight or obese increases the risk of manifold somatic diseases[7], may impact important aspects of health related quality of life [8], may affect self-esteem, and impair adherence to long term antipsychotic therapy [9,10].

WG under antipsychotic medications usually occurs shortly after starting treatment [1,9]. Whereas some data indicate that WG may reach a stable plateau after some months [10,11], this has been contested [12]. Furthermore, WG seems to be dependent not only on the drug, but also on patient related factors [4]. Whereas the best-documented individual predictor of antipsychotic induced WG has been shown to be a normal or low pre-treatment weight [13,14], some controversy remains about the link between good clinical response and WG [1,9,15].

Different mechanisms have been hypothesized as mediating WG, e.g. an increase in global caloric intake due to an appetite augmentation [16] and/or a satiety diminution [17]. On the other hand, carbohydrate craving could not be specifically related to antipsychotic induced WG [18]. Increased appetite and patient's eating behaviour seem to be better predictors of WG than the physician's selection of a specific agent [19].

If a better understanding of pharmacological and physiological basis of AP induced WG is needed, other influences on the WG phenomenon also need to be considered. Obesity can be considered as the result of interactions between physiological, environmental and behavioural factors, as well as cognitive attitudes and beliefs towards food and body shape. In a time of wide exposure to external risk factors for obesity (such as antipsychotic drugs), it may be particularly interesting to investigate personal characteristics associated with the development of obesity.

Patients having gained weight under antipsychotics regularly report on unsuccessful dietary trials as attempts to counterregulate WG [20], which suggest strong cognitive and behavioural participation to the WG phenomenon.

Whereas in general the fear of WG is likely to result in numerous rigid and inaccurate food and weight related attitudes in which the person struggles to keep control of his weight and excessively anticipates the occurrence of WG [21], this phenomenon may also be found in patients with antipsychotic-associated WG. These attitudes have been associated with restraint, which is defined as the intention to restrict food intake consciously in order to maintain body weight or to promote weight loss [22]. There might be several related behavioural strategies, which may vary in their effectiveness in long-term food reduction, and in their potential for producing disturbance of food intake regulation [5].

Furthermore, it has consistently been found, that restrained eaters show a tendency to overeat under different experimental conditions such as after food preload, after alcohol consumption and in reaction to dysphoric moods [22,23]. They were also found to increase consumption of specific food in response to related cues [24]. Several studies have also shown a correlation between dietary restrained eating and binge eating [25], which is associated with obesity in general [26,27] and also in AP induced WG [20]. Moreover, binge eating has been suggested to play a major role in WG persistence [28,29].

One can therefore hypothesize that the above-mentioned attitudes partly explain interindividual WG liabilities in patients treated with antipsychotic drugs. The purpose of the present study was to assess cognitions that could be specifically related to restrained eating in severely overweight atypical AP-treated patients with a diagnosis of schizophrenia compared to 3 comparison groups.

## Methods

### Participants

The study sample consisted in 40 outpatients with schizophrenia (DSM-IV) (19 female, mean age 33.8 ± 9.1) and 40 controls without psychiatric disability (21 female, mean age 35.5 ± 10.79). Both groups were each composed of two subgroups: one severely overweight subgroup (defined as a BMI ≥ 28), and a comparison sample (BMI < 28). All patients with schizophrenia were treated by atypical AP for 8.3 ± 6.2 years (olanzapine, clozapine, quetiapine and risperidone). Most patients were taking 1 or more comedications (alprazolam, atorvastatine, clorazepate, citalopram, esomeprazole, flurazepam, lorazepam, macrogolum, propranolol).

The recruitment procedure was as follows: the study was systematically proposed during regular consultation to outpatients with schizophrenia until reaching 20 patients in each of the BMI subgroups. In total, the study was proposed to 41 outpatients over a 6 month period. Only one patient with obesity has refused to participate in the study. Controls consisted of clinic workers, informed through local advertising. Mental disorders were excluded in non-psychiatric control subjects throw absence of actual or previous psychiatric treatment.

Informed consent was obtained from the participants before inclusion in the study.

### Design and Measures

The present survey was realized as a cross-sectional case-control study. Subject's body weight and heights were measured, and the BMI calculated as weight in kilograms divided by the square of height in meters.

Psychiatric status was assessed through a chart review, medical doctor referee and psychiatrist interview.

In order to assess the eating-related cognitive schemas, especially dysfunctional cognitions, the revised version of the Mizes Anorectic cognitive questionnaire (MAC-R) [30] was used. Although the scale name refers to anorexia, it is designed to assess cognitions for all eating disorders [30,31]. It is a 24-item self-report questionnaire that measures three dimensions of eating disorder cognitions: strict weight regulation and fear of weight gain, self-control as the basis of self-esteem, and weight and eating behaviour as the basis of approval. Each factor is 8 items in length and contributes equally to the total score [30]. The items are rated using a 5- point Likert scale, with higher scores indicating more dysfunctional cognitions. The MAC-R subscales are highly correlated to the total score and the total MAC-R score is highly correlated with the MAC [30], as well as to other eating disorder questionnaires such as the Eating Disorders Inventory [32] and the Restraint scale [33]. Both the MAC and the MAC-R have been validated in clinical and non-clinical populations [21,30], shown to assess an aspect of psychopathology that is distinct from general psychological distress [34] and finally have been found to be sensitive to the effect of cognitive-behavioural therapy [30].

### Data analyses

Descriptive statistics followed by Pearson's chi-square for categorical data and ANOVA for parametric data were performed for demographic variables and BMI.

Comparisons of MAC-R scores between groups were made using two-way analysis of covariance (ANCOVA) with weight status and psychiatric status (patients with schizophrenia vs non psychiatric subjects) as fixed factors and gender as a covariate (gender was used as a covariate as it likely have an effect on cognitions related to eating behaviors) [35]. We set alpha level at .05. All analyses were computed using the statistical software SPSS 14.0.

## Results

Concerning the four groups, we examine skewness and kurtosis statistics and their significance for MAC-R scores. Results indicate that all distribution of scores are normal."

### Characteristics of the samples

The characteristics of the 4 groups are shown in Table 1. ANOVA and post-hoc comparisons revealed no differences with regard to age. Whereas no significant differences were found with regard to gender distribution, the groups, as could easily be predicted, differed with regard to the BMI. Post-hoc tests revealed no differences between the two "BMI ≥ 28" groups and between the two "BMI < 28" groups, but significant differences between the "BMI ≥ 28" groups and the "BMI < 28" groups.

**Table 1:** Characteristics of the four groups

	**Schizophrenic patients (N = 40)**		**Non-psychiatric subjects (N = 40)**		**Statistical test**
		
	**BMI < 28**	**BMI ≥ 28**	**BMI < 28**	**BMI ≥ 28**	
	
*Age (M, SD)*	31.7 ± 9.2	36.1 ± 8.9	33.9 ± 13.0	37.2 ± 8.1	*F(3,76) *= 1.18; NS
*Women (%)*	45	50	65	40	*χ*^*2*^*(2) *= 2.8; NS
*BMI (M, SD)*	23.6 ± 2.2	32.9 ± 6.1	21.1 ± 2.5	33.8 ± 4.9	*F(3,76) *= 46.07; *p *< .05

### Cognitive distortions

Means and standard deviations of the MAC-R total score and the subscales are shown in Table 2.

**Table 2:** MAC-R scores

	Schizophrenic patients		Non psychiatric subjects	
	
	BMI < 28	BMI ≥ 28	BMI < 28	BMI ≥ 28
*Total score*	66.2 ± 14.7	72.5 ± 14.4	53.4 ± 13.8	74.1 ± 11.5
*Subscale 1: Approval*	20.0 ± 6.2	21.3 ± 6.8	16.5 ± 4.6	20.6 ± 3.5
*Subscale 2: Weight regulation*	20.5 ± 6.6	24.1 ± 7.9	14.7 ± 5.7	24.0 ± 5.8
*Subscale 3: Self-control*	26.2 ± 5.8	27.2 ± 5.8	21.7 ± 7.6	28.2 ± 6.5

#### Total score

ANCOVA results indicated that the covariate, gender, was significantly related to eating disorder cognitions (*F(1,75) *= 8.50, *p *< .05), women (69.35 ± 16.10) scoring higher than men (63.73 ± 14.91). There were significant main effects of diagnosis (*F(1,75) *= 4.33, *p *< .05) and weight group (*F(1,75) *= 24.06, *p *< .05), as well as an interaction significant effect between diagnosis and weight (*F(1,75) *= 8.29, *p *< .05), after controlling for the effect of gender. Schizophrenic patients with BMI < 28 scored higher than controls in the same range of BMI (cf. Table 2).

#### Subscale 1 (Approval)

After controlling for effect of gender, a main effect appeared for the weight factor (*F (1;75) *= 5.76; *p *< .05), but no significant effect for diagnosis and no significant interaction between diagnosis and weight.

#### Subscale 2 (Weight regulation)

The analysis revealed significant main effects of weight (*F (1;75) *= 20.89; *p *< .05] and diagnosis (*F (1;75) *= 4.39; *p *< .05], as well as an interaction significant effect between diagnosis and weight (*F(1,75) *= 4.85, *p *< .05), after controlling for the effect of gender. Schizophrenic patients with BMI < 28 scored higher than controls in the same range of BMI (cf. Table 2).

#### Subscale 3 (Self control)

The covariate, gender, was significantly related to self-control dimension (*F(1,75) *= 12.58, *p *< .05), women (27.73 ± 6.35) scoring higher than men (23.90 ± 6.81). After controlling for the effect of gender, results indicated a significant main effect of weight (*F(1,75) *= 9.65, *p *< .05), as well as a significant interaction effect between diagnosis and weight (*F(1,75) *= 6.60, *p *< .05). Similarly to previous results for total score and weight regulation subscale, schizophrenic patients with BMI < 28 scored higher than controls in the same range of BMI (cf. Table 2).

## Discussion

The aim of the present study was to assess anorectic cognitions in overweight patients with schizophrenia treated with antipsychotic drugs. Comparing these patients to patients having schizophrenia with a BMI < 28, and with non psychiatric control subjects, the following cognitive patterns emerged, regardless of gender:

Patients with schizophrenia in general scored higher on the MAC-R total scale and on the MAC-R subscale 2, the latter score representing "rigid weight regulation and fear of weight gain".

Subjects with BMI ≥ 28 as a whole scored higher on the MAC-R total score and on each subscale.

When comparing the two groups of subjects with BMI < 28, it appeared that patients with schizophrenia also scored higher on MAC-R scale and subscales 2 and 3, indicating altered "weight regulation" and "self control".

As expected [35] the group of women as a whole scored higher on the MAC-R total score and on the the subscale 3, indicating greater endorsement of self-control of weight as the basis of self-esteem.

As, in contrast to the non psychiatric subjects, even patients having schizophrenia with lower BMI show fear of weight gain and rigid weight regulation as well as altered self control, it raises the question whether these anorectic cognitions are a pre-treatment trait or whether they are induced by antipsychotic pharmacotherapy, since the distortion in these cases (still) is not associated with weight gain, by previous drug-induced weight gain or by a more modest weight gain. Lack of data on the weight gain history as well as the cross sectional design of the present study do not allow this problem to be explored. A prospective study would allow testing of the MAC-R scores as a predictor of subsequent weight gains in patients with presently low BMI.

Limitations of this study also included the choice of clinical workers with probably different social and cultural background than patients and the small size of the studied samples.

A further rationalization of this observation could be the existence of two subgroups of restrained eaters. One group of patients having schizophrenia with anorectic cognitions may be relatively successful in controlling weight, whereas the second group includes the "unsuccessful dieters".

In conclusion, the present study is, to our knowledge, the first to describe anorectic cognitive distortions in patients with schizophrenia treated with antipsychotic drugs. The present findings are consistent with those of previous studies with normal subjects and those studying patients with eating disorders [34]. As is the case in weight gain of non psychiatric subjects, the present results suggest that the cognitive distortions, as assessed by the MAC-R, may play an important role in weight gain also in patients with schizophrenia, and in weight gain associated with antipsychotic pharmacotherapy. These eating related concerns could contribute to maintenance of weight gain and thus worsening obesity. It would be interesting to know whether these subjects were experiencing binges and the relationship between restriction and loss of control over eating. Particular attention to these processes may help to improve management of antipsychotic induced weight gain.
